# Isolation and Structural Characterization of Lignin from Cotton Stalk Treated in an Ammonia Hydrothermal System

**DOI:** 10.3390/ijms131115209

**Published:** 2012-11-16

**Authors:** Sumin Kang, Lingping Xiao, Lingyan Meng, Xueming Zhang, Runcang Sun

**Affiliations:** 1 Institute of Biomass Chemistry and Technology, Beijing Forestry University, Beijing 100083, China; E-Mails: ksm0113@sina.com (S.K.); lingpingxiao@gmail.com (L.X.); menglingyan.1986@163.com (L.M.); 2 State Key Laboratory of Pulp and Paper Engineering, South China University of Technology, Guangzhou 510640, China

**Keywords:** cotton stalk, lignin, ammonia hydrothermal system, ^13^C NMR, HSQC

## Abstract

To investigate the potential for the utilization of cotton stalk, ammonia hydrothermal treatment was applied to fractionate the samples into aqueous ammonia-soluble and ammonia-insoluble portions. The ammonia-soluble portion was purified to yield lignin fractions. The lignin fractions obtained were characterized by wet chemistry (carbohydrate analysis) and spectroscopy methods (FT-IR, ^13^C and ^1^H-^13^C HSQC NMR spectroscopy) as well as gel permeation chromatography (GPC). The results showed that the cotton stalk lignin fractions were almost absent of neutral sugars (0.43%–1.29%) and had relatively low average molecular weights (1255–1746 g/mol). The lignin fractions belonged to typical G-S lignin, which was composed predominately of G-type units (59%) and noticeable amounts of S-type units (40%) together with a small amount of H-type units (~1%). Furthermore, the ammonia-extractable lignin fractions were mainly composed of β-*O*-4′ inter-unit linkages (75.6%), and small quantities of β-β′ (12.2%), together with lower amounts of β-5′ carbon-carbon linkages (7.4%) and *p*-hydroxycinnamyl alcohol end groups.

## 1. Introduction

Lignocellulosic biomass has enormous potential as a replacement for fossil fuels due to its abundance and chemical composition. In general, all kinds of lignocellulosic biomass are predominately composed of three structural polymers: cellulose, hemicelluloses, and the aromatic polymer lignin [1]. Since cellulose and hemicelluloses are sugar-based macromolecules, which amount to around 70% of the total biomass available, they can serve as raw materials for bioethanol production to replace the current source of starch-based biomass. Lignin, making up 20%–25% of plant cell walls, is a complex polymer synthesized mainly from three hydroxycinnamyl alcohols with different degree of methoxylation [2,3]. Each of these monolignols gives rise to a different type of lignin unit namely; *p*-hydroxyphenyl (H), guaiacyl (G), and syringyl (S) units, when incorporated into the polymer [4]. The lignin polymer provides mechanical support to the plant and plays a role in protecting plants against pathogens [5].

Until now, the characteristics of lignin have not yet been completely elucidated due to its compositional and structural complexity. The amount and composition of lignin vary among taxa, cell types, and individual cell wall layers. Softwood lignin consists almost exclusively of G-type lignin, hardwood lignin consists of G and S-type units (H units being minor components), while gramineae has all the three [6]. In addition, different levels of regulations including (a) metabolic control, (b) regulation of individual enzymes in the biosynthetic pathway, and (c) regulation of gene expression also affect the lignin content, quality, and distribution [7,8]. Through these methods, the designed plants either deposit less lignin or produce lignins that are more amenable to chemical degradation [9–11]. The three major C_6_–C_3_ (phenylpropanoid) units of lignin are connected by ether and carbon-carbon linkages, such as β-*O*-4′, 4-*O*-5′, β-β′, β-1′, β-5′, and 5-5′ [12]. In addition to the various types of bonds present within the lignin itself, there are associations between lignin and polysaccharides [13], forming a lignin-carbohydrate complex with benzyl-ether, benzyl-ester, and phenyl-glycoside bonds [14]. Currently, vast quantities of lignin are burnt in paper mills to produce process heat, power, steam and to recover pulping chemicals [15]. If ethanol industries based on lignocellulosic biomass grow in the near future, the integration of transition of materials from lignin and overall heat balance need to be considered [16]. Therefore, studying the isolation, structural characterization and potential utilization of lignin appears to be crucial, and has become very intense in industries such as, agriculture and the paper industry [17].

The tight physical binding and chemical linkages between lignin and cell wall polysaccharides make it difficult to isolate lignin in an unaltered form. The isolation of lignin with high yields and minimal chemical modification is still a major challenge for structural characterization of lignin [18]. Various standard methods have been traditionally used to isolate lignin from wood. A mild and widely used separation method was established by Björkman [19], which is based on extensive grinding of plant material followed by extraction with dioxane/water. The milled wood lignin (MWL) is considered to be representative of the native lignin structure and has been extensively used in the elucidation of native lignin structure. Furthermore, enhancement on the lignin isolation based on hydrothermal pretreatment has been made as reported in previous studies [20–22]. The results showed that steam hydrolysis resulted in an extensive liberation of phenolic groups, and undoubtedly this was primarily due to cleavage of the aryl ether bonds in β-*O*-4′ structures, whereas the contents of C–C condensed structures increased during steam treatment [22]. Sugar degradation products (such as furfural) reacted with lignin, which increased the acid insoluble component of the plant [23]. In addition, syringyl units situated mainly in S_2_ layers in hardwood were more susceptible to hydrothermal degradation than guaiacyl units [24,25]. It was also shown that the MWL isolated from the hydrothermal treated solid residue was more condensed and had a lower molecular weight than the MWL isolated from the untreated material [26]. Lignin reaction kinetics during the hydrothermal treatment has also been reported by Zhang *et al.*[27]. The results indicated that the lignin degradation mechanism consisted of a two phase reactions for the hydrothermal treatment: first, a very fast reaction phase where lignin was degraded into the soluble fragments and then a slower reaction phase where the soluble fragments reacted with one another via recondensation. Recently, ammonia has been considered to be a desirable pretreatment reagent [28]. This is because it was effective in improving cellulose digestion and had high reaction selectivity towards lignin over carbohydrates. It has been demonstrated that the reactions of aqueous ammonia with lignin are the cleavage of C–O–C bonds in lignin and ether and ester bonds in the lignin-carbohydrate complex (LCC). In addition, its high volatility makes it easy to recover and reuse [29].

Cotton stalks, the lignocellulosic by-products from cotton production, have a considerable economic and ecological importance. In China, it is estimated that more than 20 million tons (dry weight) of cotton stalk are generated annually [30]. However, most of the cotton stalk is burned off, or even sometimes the stalk is left in the ground to decay, without being fully utilized. As a lignocellulosic feedstock with high potential, the utilization efficiency of this lignocellulosic biomass is highly dependent on its structural properties, such as the relative content, composition, accessibility, and reactivity of the three cell wall components. Particularly, the lignin content and its composition in terms of guaiacyl (G), syringyl (S), and *p*-hydroxyphenyl (H) moieties as well as the nature of the different inter-unit linkages are important factors affecting delignification rate, hydrolysis rate, and chemical consumption [31]. In this study, in order to investigate the potential for the utilization of cotton stalk, separation of lignin components from cotton stalk based on ammonia hydrothermal treatment was investigated. Under the alkalinity of ammonia, part of the linkages between lignin and carbohydrates are likely to be broken, so as to achieve the purpose of lignin’s separation. In addition, MWL from the same material was also isolated as a comparison. The structure of the lignin fractions obtained were elucidated by ion chromatography, gel permeation chromatography (GPC), Fourier transform infrared (FT-IR) spectroscopy, ^13^C nuclear magnetic resonance (^13^C NMR) spectroscopy and ^1^H-^13^C correlation two-dimensional nuclear magnetic resonance (^1^H-^13^C 2D NMR) spectroscopy.

## 2. Results and Discussion

### 2.1. Purity of Lignin Fractions

To verify the purity of the lignin fractions, the compositions of the bound neutral sugars in these lignin fractions were determined and the results are given in Table 1. The sugar contents of the lignin fractions ranged from 0.43% to 1.29%, which were lower than that of MWL. Glucose was the dominant sugar component (0.36%–1.21%), while trace amounts of rhamnose, arabinose, galactose, xylose, and mannose were also identified in these lignin fractions. High purity of the lignin fraction is important because ammonia had high reaction selectivity towards lignin over carbohydrates [28]. It was noted that the carbohydrate contamination in lignin fraction L_5_ was higher in comparison with lignin fractions of L_3_ and L_4_. This was likely caused by the release of more amounts of carbohydrates due to the longer extraction time in lignin fraction L_5_. The carbohydrate contamination in the lignin fractions from the ammonia hydrothermal separations was lower as compared to a total dissolution process (9.1%) [32]. This demonstrated the potential of ammonia hydrothermal treatment for high purity lignin separation.

### 2.2. Molecular Weight Distribution

**Figure 1:**
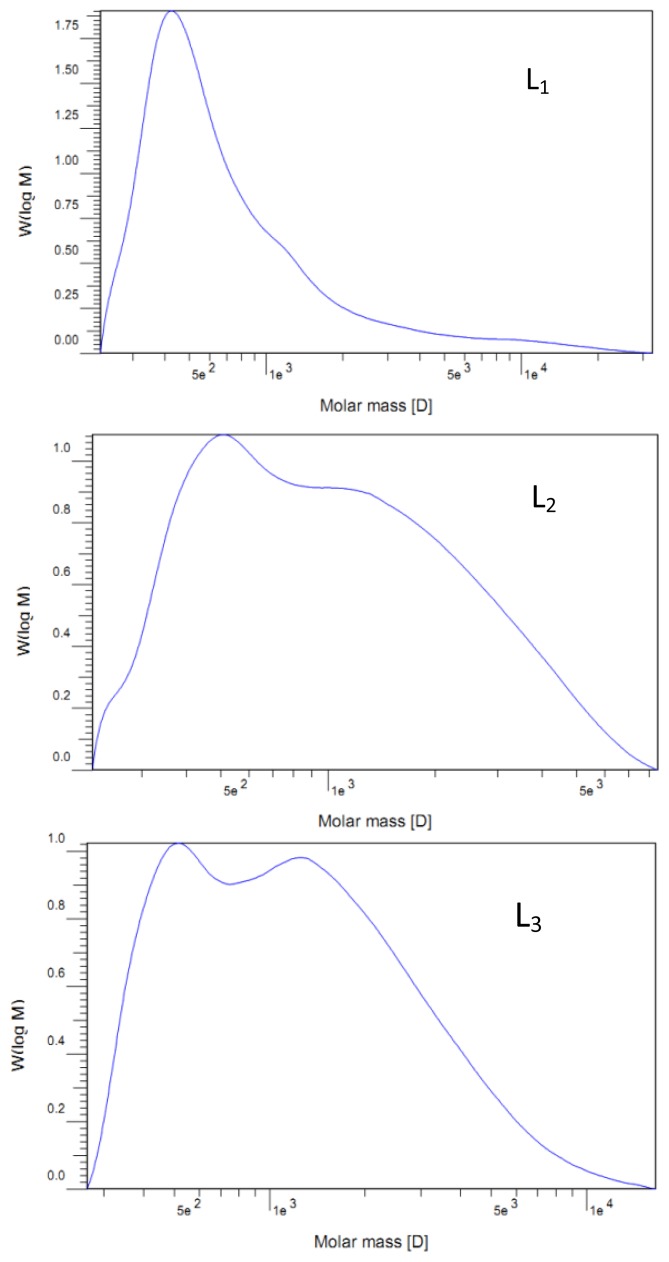

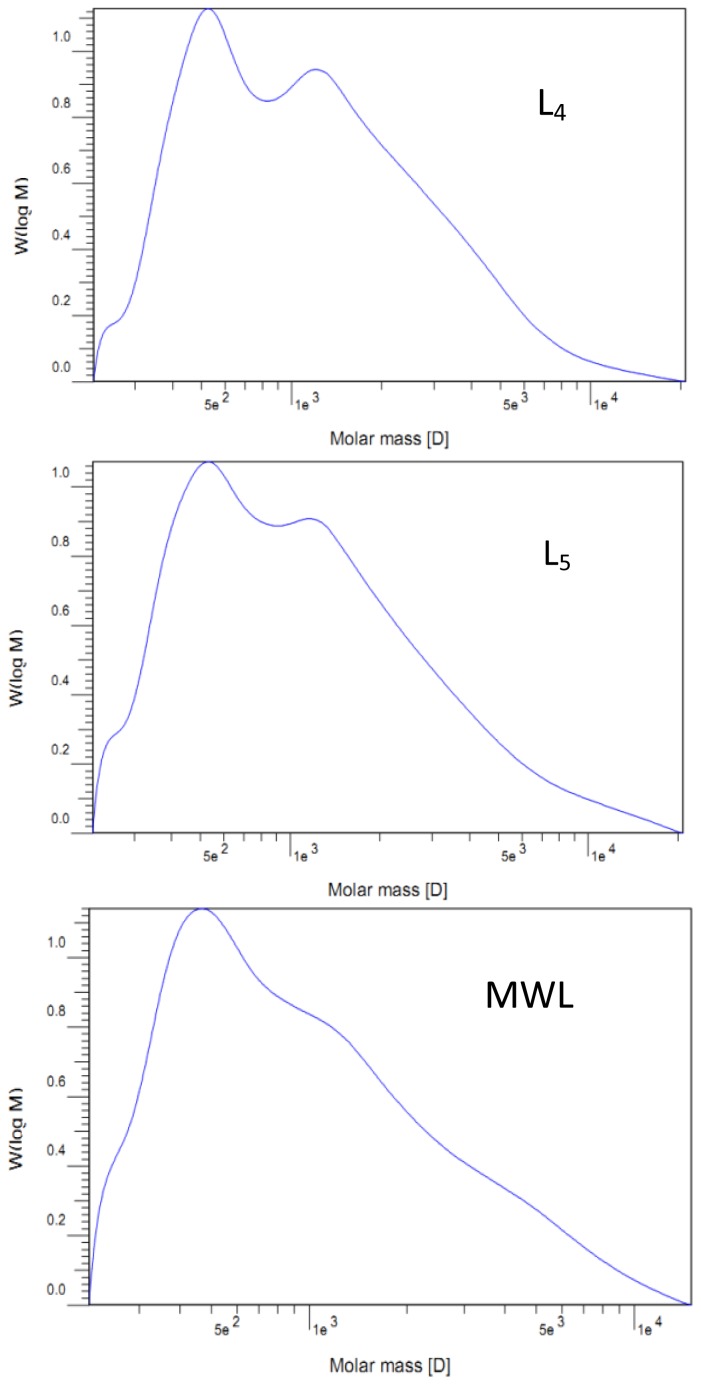
Molecular weight distribution curves of the lignin fractions.

The question as to whether the ammonia hydrothermal treatment had caused lignin depolymerization was addressed by investigating the GPC elution curves for all the lignin fractions. The values of the weight-average (*M*_w_) and number-average (*M_n_*) molecular weight and the polydispersity (*M*_w_*/M_n_*) of all lignin preparations are listed in Table 2. In addition, molecular weight distribution curves are also shown in Figure 1.

The weight-average molecular weights were between 1250 and 1740 g/mol, and number-average molecular weights ranged from 560 to 890 g/mol, as shown in Table 2. The molecular-average weights showed no significant difference between lignin fractions. The relatively low molecular weights of the lignin fractions demonstrated that the extractions with aqueous ammonia could only separate the low molecular weight lignin fragments due to the weak alkalinity. The results obtained in this study have also been verified by corresponding data from literature, in which the molecular weight of lignin samples extracted by dilute alkali from straw was also relatively low [33,34]. Furthermore, molecular weight distribution curves illustrate the structural features of the lignin fractions to a certain degree. As shown in Figure 1, lignin fractions L_2_, L_3_, L_4_, and L_5_, isolated with reaction time of 4, 6, 8, and 10 h, exhibited bimodal molecular weight distributions as compared with lignin fraction L_1_.

### 2.3. FT-IR Spectra

**Figure 2:**
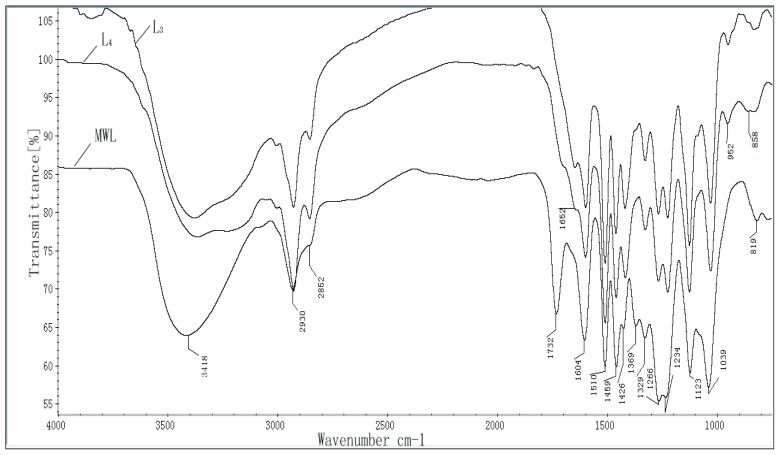
Fourier transform infrared (FT-IR) spectra of lignin preparations of L_3_, L_4_, and milled wood lignin (MWL) from dewaxed cotton stalk.

FT-IR spectra of lignin fractions of L_3_, L_4_ (as the representativeness), and MWL are shown in Figure 2, and the main assignments of FT-IR bands are summarized in Table 3. As shown in Figure 2, lignin fractions of L_3_, L_4_, and MWL have absorption peaks at 1604, 1510, 1459 and 1426 cm^−1^ corresponding to the vibration absorption of aromatic units [35]. The peak intensities for aromatic skeleton vibrations of L_3_ and L_4_ were rather similar, indicating that there was no drastic change of the structure of lignin during the treatment process. A wide absorption at 3418 cm^−1^ is assigned to the O–H stretching vibration in aromatic and aliphatic O–H groups, and the absorption bands at 2930 and 2852 cm^−1^ are assigned to asymmetric and symmetrical vibrations of saturated CH_2_ in side chain of lignin, respectively. It should be noted that the intensity of the band at 1732 cm^−1^ assigned to unconjugated carbonyl groups was observed in spectrum of MWL while it occurred as a shoulder in the spectra of L_3_ and L_4_, which implied that the carbonyl groups in lignin fractions L_3_ and L_4_ had been removed during the ammonia hydrothermal treatment. The absorption peaks at 1329 cm^−1^ is assigned to syringyl units and the peak at 1266 cm^−1^ is assigned to guaiacyl ring units [36]. The strong intensity of the bands at 1123 and 1039 cm^−1^ corresponds to the aromatic C–H in-plain deformation. Aromatic C–H out of plane vibration was exhibited at 819 cm^−1^.

According to the lignin classification system for infrared spectra by Faix [36], it could be concluded that lignin fractions L_3_ and L_4_ were belonged to the typical G-S lignin types due to the following characteristics; firstly the intensity of the band at 1510 cm^−1^ is higher than that of the band at 1459 cm^−1^, secondly there is the strong signal at 1123 cm^−1^ and lastly, the absorption peak at 1329 cm^−1^ is weaker than that at 1266 cm^−1^.

### 2.4. ^13^C-NMR Spectra

**Figure 3:**
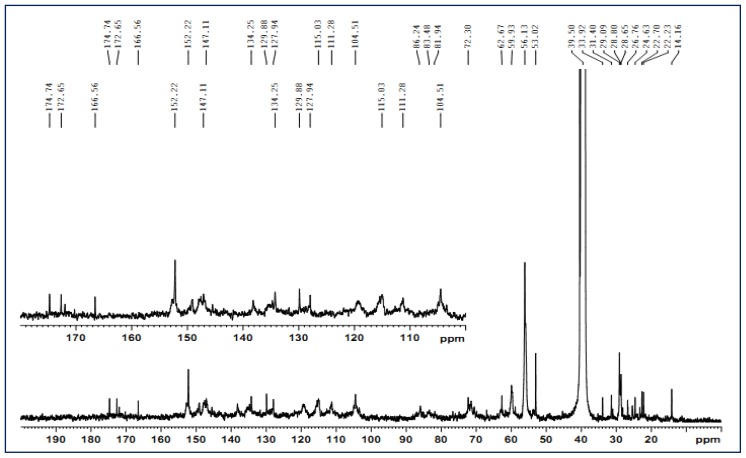
^13^C nuclear magnetic resonance (^13^C NMR) spectrum of lignin fraction L_3_.

As a powerful method to determine the structural features of lignin, the analytical techniques of ^13^C-NMR has been widely applied for the identification of lignin structure. To further investigate the lignin structural features, lignin fraction L_3_ was investigated by ^13^C NMR spectroscopy. The spectrum is shown in Figure 3, and the characteristic signals of the lignin fraction are shown in Table 4. From the ^13^C-NMR spectrum, some symmetrical signals, which generally believed to be caused by lignin molecules, were qualitatively detected. The weak signal at 174.7 ppm was attributed to carbon in carbonyl groups from side-chain carboxyl group [37], which was confirmed by the presence of a signal at 166.6 ppm corresponding to α carboxylic carbon [38]. As expected, it was almost absence of typical polysaccharide signals between 100 and 60 ppm except the signal at 62.7 ppm (C-5 in xylose non-reducing end unit) which confirmed that only traces of associated polysaccharides existed in the lignin fraction. This qualitative observation was in good agreement with the previously described results of sugar analysis (Table 1). In addition, the syringyl, guaiacyl, and *p*-hydroxyphenyl aromatic carbons were detected qualitatively and shown in the aromatic area of the ^13^C-NMR spectra from 104.5 to 166.6 ppm. From the comparison of the absorption signal intensities of guaiacyl, syringyl, and *p*-hydroxyphenyl units of lignin, it could be concluded that the separated lignin contains a high content of guaiacyl units and a considerable amount of syringyl units and a few amount of *p*-hydroxyphenyl units.

Qualitative ^13^C-NMR analysis of lignin preparation also allowed for the quantification of β-*O*-4′ aryl ether structures. As shown in Figure 3, the signals at 86.2 (C-β in S β-*O*-4′ erythro), 72.3 (C-α in β-*O*-4′ G and S erythro), and 59.9 ppm (C-γ in β-*O*-4′ G and S threo and erythro) belonged to the resonances of C-β, C-α, and C-γ in β-*O*-4′ linkages, respectively. Accordingly, it could be concluded that large amounts of β-aryl ether structures were still preserved in lignin fraction L_3_ after being treated in the ammonia hydrothermal system. In addition, the common carbon-carbon linkages, such as β-β′ (C-α in β-β′ units, 83.5 ppm) and β-5′ (C-β in β-5′ units, 53.0 ppm; C-γ in β-5′ units, 62.7 ppm) were also qualitatively detected. The signals for the γ-methyl, α and β-methylene groups in *n*-propyl side chains of the lignin fraction appeared in the spectrum between 14.2 and 33.9 ppm. Moreover, the very strong signal at 56.1 ppm is assigned to –OCH_3_ in syringyl and guaiacyl units. To sum up, the lignin fraction separated from the cotton stalk based on the treatment with ammonia hydrothermal system consisted mainly of the β-*O*-4′ ether bond, combined with a small amount of C–C bonds (β-β′, β-5′).

### 2.5. 2D HSQC NMR

**Figure 4:**
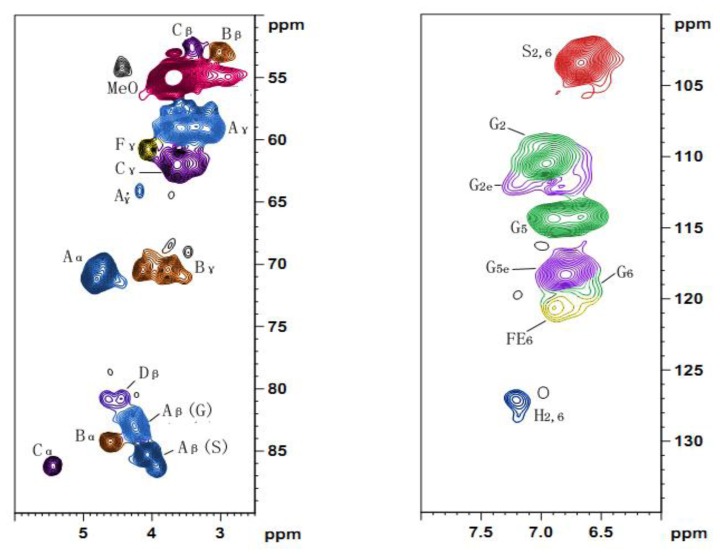
2D the heteronuclear single quantum correlation (HSQC) NMR spectrum of lignin fraction L_3_.

**Figure 5:**
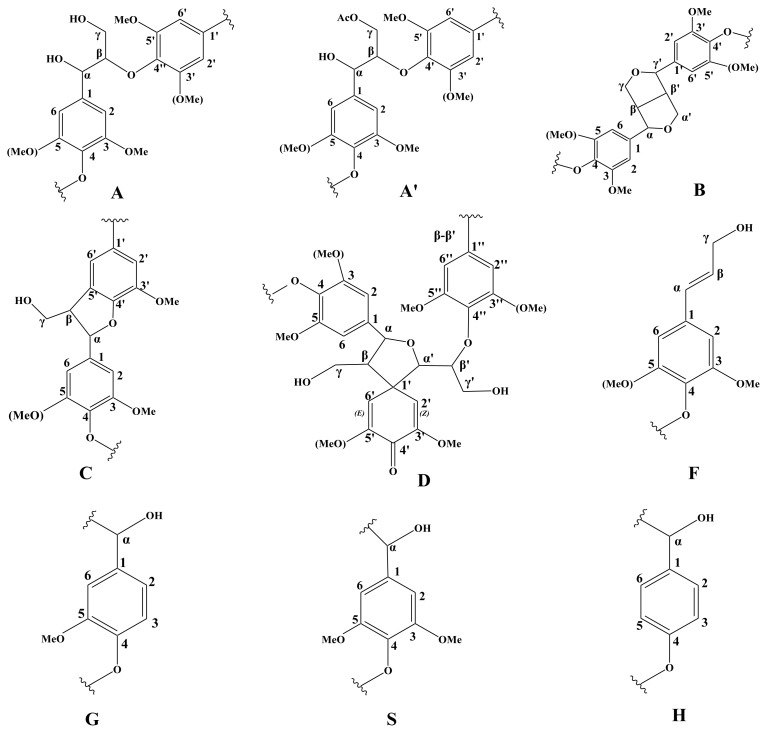
Main structures presented in ammonia-extractable lignin. (**A**) β-*O*-4′ linkages; (**A′**) γ-acetylated γ-*O*-4′ substructures; (**B**) resinol structures formed by β-β′/α-*O*-γ′/γ-*O*-α′ linkages; (**C**) phenylcoumarane structures formed by β-5′/α-*O*-4′ linkages; (**D**) spirodienone structures formed by β-1′ linkages; (**F**) *p*-hydroxycinnamyl alcohol end groups; (**G**) guaiacyl unit; (**S**) syringyl unit; (**H**) *p*-hydroxyphenyl unit.

To obtain a further comprehensive structural characterization of cotton stalk lignin, lignin fraction L_3_ was subjected to 2D NMR analysis. The HSQC NMR spectrum (Figure 4) of the ammonia-extractable lignin fraction exhibited three regions, which are the aliphatic (δ_C_/δ_H_ 10–40/0.5–2.5 ppm), side chain (δ_C_/δ_H_ 50–95/2.5–6.0), and aromatic ^13^C-^1^H correlations (δ_C_/δ_H_ 95–150/5.5–8.0) regions. The assignments of the main cross-signals in the HSQC spectra are listed in Table 5 and the main substructures presented are depicted in Figure 5.

Common structures from the various inter-unit linkage types were detected and shown in the side-chain region of the spectrum (Figure 4). For instance, the α-, β- and γ-position of β-*O*-4′ linkages are presented at δ_C_/δ_H_ 71.1/4.76, 83.2/4.23 and 59.1/3.26-3.60 ppm, respectively [39]. Moreover, the C_β_–H_β_ correlations corresponding to the erythro and threo forms of the S-type β-*O*-4′ substructures could be distinguished at δ_C_/δ_H_ 85.5/4.07 and 86.1/3.91 ppm [40,41]. A predominance of the erythro over the threo diasteroisomers was also observed in β-*O*-4′ structures, which was in accordance with the previous results that lignin from angiosperms contains higher proportion of erythro forms than threo forms in β-*O*-4′ units [39]. Other various inter-unit linkages were also observed in substantial amounts. Strong signals for resinol structures (β-β′/α-*O*-γ′/γ-*O*-α′ linkages, B) were observed in the spectra, with their C–H correlations for α-, β-C positions at δ_C_/δ_H_ 84.3/4.61, 52.9/3.02 ppm, respectively and double γ-C positions at δ_C_/δ_H_ 70.4/3.76 and 4.13 ppm. The phenylcoumarans (β-5′, C) units were also identified from the spectra, and the signals for their C_α_–H_α_, C_β_–H_β_ and C_γ_–H_γ_ correlations are observed at δ_C_/δ_H_ 86.2/5.44, 52.6/3.41, and 62.0/3.64 ppm, respectively. Moreover, trace amounts of spirodienone unit (D) and *p*-hydroxycinnamyl alcohol end groups (F) were also observed by their C_β_–H_β_ and C_γ_–H_γ_ correlations at δ_C_/δ_H_ 80.9/4.45 and 60.7/4.06 ppm, respectively.

In the aromatic region of the HSQC spectrum, the main cross-signals were attributed to the substituted phenyl rings of the different lignin units. Signals from guaiacyl (G), syringyl (S), and *p*-hydroxyphenyl (H) units were all qualitatively detected. G lignin units showed different correlations at C_2_–H_2_ (δ_C_/δ_H_ 110.5/6.94 ppm), C_5_–H_5_ (δ_C_/δ_H_ 114.3/6.67 and 6.90 ppm), and C_6_–H_6_ (δ_C_/δ_H_ 119.5/6.59 ppm). The multiple C_5_–H_5_ signals revealed some heterogeneity among the G units which was probably due to different substituents of phenolic or etherified structure at C_4_[39]. Trace amounts of *p*-hydroxyphenyl (H) units were observed from C_2,6_–H_2,6_ correlations at δ_C_/δ_H_ 127.2/7.20 ppm, indicating that there was a minor amount of H units in the ammonia-extractable lignin.

The relative abundances of main inter-unit linkages, the percentage of γ-acetylation, relative molar composition of S, G, H units as well as the S/G molar ratios were semi-quantitatively estimated from the HSQC spectrum and data are listed in Table 6. Clearly, the β-*O*-4′ aryl ether linkages (75.6%) occupied the predominance of side chains inter-unit linkages in ammonia-extractable lignin, followed by β-β′ resinol-type linkages (12.2%), β-5′ phenylcoumaran (7.4%), and *p*-hydroxycinnamyl alcohol end groups (4.9%). The high percentage of β-*O*-4′ aryl ether linkages gave an explanation for the increased difficulty to extract lignin from cotton stalk. The ratio of S/G was 0.7 (S < G), in agreement with the result of ^13^C NMR and FT-IR spectra analysis. Therefore, it could be concluded that the lignin fraction obtained from cotton stalk was composed predominately of G-type units, together with a substantial amount of S-type units and a relatively low amount of H-type units.

## 3. Experimental Section

### 3.1. Materials

Cotton stalk was obtained from Hebei province, China. It was dried in an oven at 50 °C and cut into small pieces. The cotton stalk was then ground and screened to obtain particles ranging from 40 to 80 mesh. The chemical composition of the stalks was: cellulose 34.70%, hemicelluloses 38.62%, lignin 20.99%, and ash 1.90%. A simple extraction procedure is available that solubilizes essentially all the lipid, lignin, and hemicellulose, leaving the cellulose fibers intact. The milled powder was extracted with 90% (*v*/*v*) acetone/water in a Soxhlet apparatus for 24 h. The extracted stalk powder was dried under vacuum for several days. For determining the content of cellulose, the extractive-free cotton stalk was delignified using sodium chlorite under pH 3.8–4.2 adjusted by acetic acid for 2 h at 70 °C. Samples were then washed extensively with distilled water until the filtrate pH was neutral. The obtained holocellulose was then extracted with 10% potassium hydroxide at room temperature for 16 h with the vigorous stirring. In this procedure, hemicelluloses were dissolved in alkaline solution. The cellulose fibers could be obtained by filtration the suspension with 100% polyester cloth and washed thoroughly with distilled water until pH = 7. Finally, the cellulosic preparation was oven-dried at 50 °C overnight, and kept in a desiccator for the further experiment. Ash was determined according to TAPPI standard method T211 om-93. Klason lignin was determined according to the TAPPI standard method T-222 om-98. All chemicals used were analytical grade and standard sugars were purchased from Sigma-Aldrich Company (Beijing).

### 3.2. Isolation and Purification of Lignin Fractions

**Figure 6:**
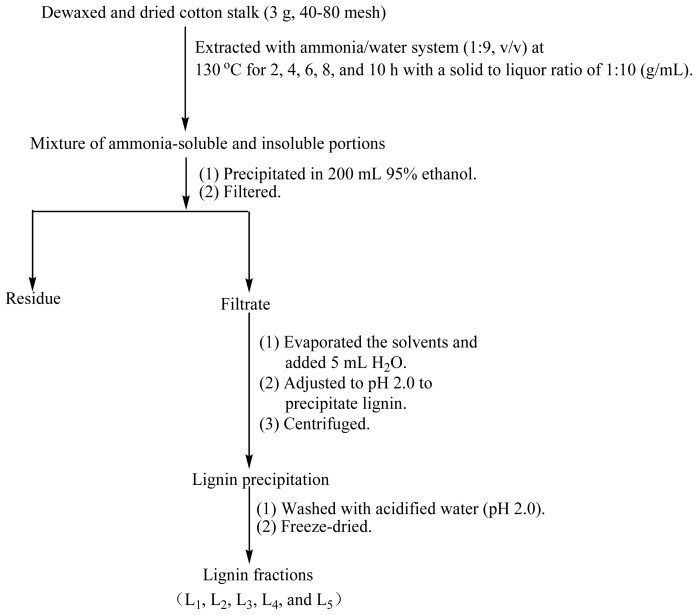
Scheme for separation of lignin fractions from cotton stalk based on ammonia hydrothermal system treatment.

Figure 6 shows the fractional separation sequence for lignin preparations based on ammonia hydrothermal system treatment. It has been shown that furfural increased sharply beyond extraction temperature at 160 °C in hot water from woodchips [42]. Therefore, the extraction conditions including temperature, solid to liquid ratio, ammonia concentration were set as follows based on pretreatment conditions in previously published papers [42,43]. Three grams of the prepared cotton stalk and 30 mL of 10% (*v*/*v*) ammonia was mixed and incubated in a Parr reactor at 130 °C for 2, 4, 6, 8, and 10 h, respectively. The brown solution was transferred into 200 mL of 95% ethanol under continuous agitation to precipitate the carbohydrates. The suspension was then filtered through filter paper using a Buchner funnel under reduced pressure. The lignin-containing solution was combined with the washing solution and concentrated under reduced pressure, followed by an addition of 5 mL deionized water. The lignin was precipitated at pH 1.5–2.0 by dropwise addition of 1 M HCl and then centrifuged at 4000 rpm for 20 min. The lignin precipitation was washed twice with acidified water (pH 2.0), centrifuged under the conditions described above and freeze-dried. Milled wood lignin (MWL) was also prepared from the same material referring to the literature for comparison [20]. The procedures are as follows: the extractive-free cotton stalk powder was subjected to milling in a planetary ball mill using ZrO_2_ balls for 10 h, followed by dioxane (96% *v*/*v*) extraction. After that, the dioxane was removed by evaporation. The obtained crude MWL was then dissolved in 90% acetic acid and precipitated drop-wise into water. The precipitate was separated by centrifugation and was dried in a vacuum oven at 35 °C. The lignin extraction efficiency from cotton stalk dissolved in the ammonia hydrothermal system at the reaction time of 2, 4, 6, 8, and 10 h showed that the yield of five lignin fractions and MWL were 14.6, 15.2, 15.7, 16.2, 16.6, and 8.2% (Klason lignin), respectively. These lignin fractions treated with ammonia hydrothermal system at the reaction time of 2, 4, 6, 8, and 10 h were denoted as L_1_, L_2_, L_3_, L_4_, and L_5_, respectively.

### 3.3. Sugar Analysis

The monosaccharide associated with lignin fractions was determined by hydrolysis with dilute sulfuric acid. Lignin samples weighing 4–6 mg were hydrolyzed with 1.475 mL of 6.1% H_2_SO_4_ for 2.5 h at 105 °C. Subsequently, the mixture was filtered through a 0.22 μm PTFE filter to remove unhydrolyzed residues, and the filtrate containing the liberated neutral sugars was diluted 50-fold and analyzed by high-performance anion exchange chromatography (HPAEC) system (Dionex ICS3000, U.S.) with a pulsed amperometric detector and an ion exchange Carbopac PA-1 column (4 × 250 mm).

### 3.4. Determination of Molecular Weight

The molecular weights (*M*_w_ and *M_n_*) of the lignin fractions were determined by gel permeation chromatography (GPC, Agilent1200, USA) with a refraction index detector (RID) on a PL-gel 10 μm Mixed-B 7.5 mm ID column, calibrated with PL polystyrene standards. The samples were dissolved with tetrahydrofuran (THF) with a concentration of 0.2%, and 20 μL aliquots of the lignin solutions were injected. The column was operated at ambient temperature and eluted with THF at a flow rate of 1.0 mL/min.

### 3.5. FT-IR Spectral Characterization

Fourier transform infrared (FT-IR) spectra of the lignin fractions were obtained from an FT-IR spectrophotometer (Tensor 27, Bruker, Germany) using a KBr disk containing 1% finely ground samples. Thirty-two scans were taken of each sample, recorded from 4000 to 400 cm^−1^ at a resolution of 2 cm^−1^ in the transmission mode.

### 3.6. Nuclear Magnetic Resonance Spectra

The solution-state ^13^C-NMR spectroscopy was carried out on a Bruker AV III 400 MHz NMR spectrometer. The sample (80 mg) was dissolved in 0.5 mL of dimethyl sulfoxide-*d*_6_ (DMSO, 99.8%), and the spectrum was recorded at 25 °C after 30,000 scans. A 30° pulse flipping angle, a 9.2 μs pulse width, a 1.89 s delay time between scans, a 1.63 s acquisition time and a 2 s relaxation time were used.

The 2D HSQC spectrum was acquired on the same NMR spectrometer in the HSQC GE experiment mode. The spectral widths for the heteronuclear single quantum correlation (HSQC) (semi-quantitative mode) were 5000 and 20,000 Hz for the ^1^H- and ^13^C-dimensions, respectively. The number of collected complex points was 1024 for the ^1^H- dimension with a recycle delay (*d*_1_) of 5 s, the number of transients for the HSQC spectrum was 128, and 256 time increments were recorded in the ^13^C-dimension. The ^1^J_C–H_ used was 146 Hz. Prior to Fourier transformation, the data matrixes were zero filled up to 1024 points in the ^13^C-dimension. Data processing was performed using standard Bruker Topspin-NMR software.

A semi-quantitative analysis of the HSQC cross-signal intensities was also performed. Since the cross-signal intensity depended on the particular ^1^J_C–H_ value, as well as on the T_2_ relaxation time, a direct analysis of the intensities was impossible. Therefore, the integration of the cross-signals was performed separately for the different regions of the HSQC spectra, which contained signals that corresponded to chemically analogous carbon-proton pairs (in similar samples). For these signals, the ^1^J_C–H_ coupling value was relatively similar and was used semi-quantitatively to estimate the relative abundance of the different lignin fractions. The inter-unit linkages of the aliphatic oxygenated region were estimated from C_α_–H_α_ correlations to avoid possible interference from homo-nuclear ^1^H–^1^H couplings, and the relative abundance of side chains involved in inter-unit linkages and terminal structures was calculated. The C–H correlations from S and G type units in the aromatic region were used to estimate the S/G ratio of lignin and the percentage of oxidized units. Moreover, the volume integrals were corrected for proton numbers to obtain an accurate S/G ratio.

## 4. Conclusions

Ammonia-extractable lignin was fractionated and characterized after ammonia hydrothermal treatment. The results showed that lignin fractions obtained from cotton stalk were typical of G-S lignin with low amounts of H units. The linkages in the ammonia-extractable lignin fractions were composed predominately of β-*O*-4′ inter-unit linkages, together with small quantities of β-β′ and low amounts of β-5′ carbon-carbon linkages. Overall, the complete characterization of the ammonia-extractable lignin fraction is important for the understanding of the lignin structural features as well as for potential utilization of cotton stalk for biomaterials. Although further experiments are required to improve the aqueous ammonia concentration to extract the lignin component more efficiently, this study has clarified that ammonia hydrothermal system is applicable for fractionation of lignin from cotton stalk.

## Figures and Tables

**Table 1:** The content of neutral sugars (% dry weight, *w*/*w*) in lignin fractions separated using ammonia hydrothermal system from dewaxed cotton stalk.

Sugars (%)	Lignin fractions a					

	L_1_	L_2_	L_3_	L_4_	L_5_	MWL
Rhamnose	ND b	ND	0.02	0.01	0.03	0.09
Arabinose	ND	ND	0.02	0.01	0.05	0.09
Galactose	0.03	0.01	0.07	0.04	0.05	0.09
Glucose	1.18	1.21	0.70	0.36	0.83	2.04
Xylose	0.05	0.03	0.12	0.01	0.09	4.43
Mannose	0.03	ND	0.05	ND	ND	ND
Total	1.29	1.25	0.98	0.43	1.06	6.74

a L_1_, L_2_, L_3_, L_4_, and L_5_ represent the lignin fractions isolated based on ammonia hydrothermal treatment at different reaction time of 2, 4, 6, 8, and 10 h;

b ND = not detected.

**Table 2:** Weight-average (*M*_w_) and number-average (*M_n_*) molecular weights and polydispersity (*M*_w_/*M^n^*) of the lignin fractions separated using ammonia hydrothermal system.

	Lignin fractions a					

	L_1_	L_2_	L_3_	L_4_	L_5_	MWL
*M*_w_	1250	1390	1700	1690	1740	1520
*M_n_*	560	760	890	830	790	700
*M*_w_/*M_n_*	2.23	1.83	1.91	2.04	2.20	2.17

a Corresponding to the lignin fractions in Table 1.

**Table 3:** Main assignments of ammonia-extractable lignin fractions in FT-IR spectra.

Absorption band (cm^−1^)	Assignment
3418	O–H stretching vibration in aromatic and aliphatic OH groups
2930	C–H stretching vibrations in OCH_3_
2930, 2852	C–H asymmetric and symmetrical vibrations in saturated CH_2_
1732	Unconjugated carbonyl groups
1652	Carbonyl stretching in para-substituted ketones or aryl aldehydes
1604	Aromatic ring vibrations and C=O stretching (S > G)
1510	Aromatic skeletal vibrations (G > S)
1459	Asymmetric C–H deformations (in CH_3_ and –CH_2_–)
1426	Aromatic skeletal vibrations combined with C–H in plane deform
1360	COO-asymmetric and symmetrical vibrations in carboxylate groups
1329	Syringyl units
1266	Guaiacyl units
1234	C–C, C–O, and C=O stretch (G condensed > G etherified)
1123	Aromatic in-plane C–H bending (typical for S units)
1039	Aromatic C–H in-plane deformation (G > S units)
952	–HC=CH-out-of-plane deform. (trans)
858	Aromatic C–H out of bending
819	C–H out-of-plane in position 2 and 6 of S units, and in all positions of H units

**Table 4:** Chemical shift value (δ, ppm), intensity, and signal assignment of the ammonia-extractable lignin fraction L_3_.

PPM	Intensity	Assignment	PPM	Intensity	Assignment
174.7	M	Aliphatic carboxyl carbon	72.3	W	C-α, G and S units
172.7	M	As above	62.7	M	C-5, xylose unit
166.6	W	C-α, carboxylic carbon	59.9	S	C-γ, G and S units
152.2	M	C-3/C-5, S units	56.1	S	OCH_3_, G and S units
134.3	W	C-1, S units etherified; C-1, G units etherified	53.02	VW	C-β, β-5′ units
129.9	W	C-1, G units	33.9	W	CH_3_ in ketones or in aliphatic side chain
127.9	W	C-2/C-6, H units	31.4	W	As above
115.0	M	C-5, G units	29.1	M	CH_2_ in aliphatic side chain
111.3	M	C-2, G units	25.3	W	CH_3_ or CH_2_ group in side chains
104.5	M	C-2/C-6, S units	24.6	M	As above
86.2	W	C-β, β-*O*-4′	22.7	W	As above
83.5	W	C-α, β-β′	14.2	W	γ-CH_3_ in *n*-propyl side chain

Intensity abbreviations: G, guaiacyl; S, syringyl; H, *p*-hydroxyphenyl; W, weak; M, medium; S, strong; VW, very weak.

**Table 5:** Assignments of ^13^C-^1^H correlation signals in the HSQC NMR spectrum of the ammonia-extractable lignin fraction L_3_ from the cotton stalk.

Lables	δ_C_/δ_H_	Assignment
C_β_	52.6/3.41	C_β_–H_β_ in phenylcoumaran substructures (C)
B_β_	52.9/3.02	C_β_–H_β_ in β-β′ (resinol) substructures (B)
MeO	54.9/3.70	C–H in methoxyls
A_γ_	59.1/3.26 and 3.60	C_γ_–H_γ_ in β-*O*-4′ substructures (A)
F_γ_	60.7/4.06	C_γ_–H_γ_ in *p*-hydroxycinnamyl alcohol end groups (F)
C_γ_	62.0/3.64	C_γ_–H_γ_ in phenylcoumaran substructures (C)
A′_γ_	64.1/4.18	C_γ_–H_γ_ in γ-acylated β-*O*-4′ substructures (A′ and A″)
B_γ_	70.4/3.76 and 4.13	C_γ_–H_γ_ in β-β′ resinol substructures (B)
A_α_	71.1/4.76	C_α_–H_α_ in β-*O*-4′ substructures linked to an S units (A)
D_β_*_′_*	80.9/4.45	C_β′_–H_β′_ in spirodienone substructures (D)
A_β(G)_	83.2/4.23	C_β_–H_β_ in β-*O*-4′ substructures linked to G and H units (A)
B_α_	84.3/4.61	Cα–Hα in β-β′ (resinol) substructures (B)
A_β(S)_	85.5/4.07	C_β_–H_β_ in β-*O*-4′ substructures linked to S units (A)
A_β(S)_	86.1/3.91	C_β_–H_β_ in β-*O*-4′ substructures linked to S units (A)
C_α_	86.2/5.44	C_α_–H_α_ in phenylcoumaran substructures (C)
S_2,6_	103.3/6.66	C_2,6_–H_2,6_ in etherified S units (S)
G_2_	110.5/6.94	C_2_–H_2_ in G units (G)
G_2e_	112.1/7.21	C_2_–H_2_ in etherified G units (G)
G_5_	114.3/6.67 and 6.90	C_5_–H_5_ in G units (G)
G_5e_	118.3/6.79	C_5_–H_5_ in etherified G units (G)
G_6_	119.5/6.59	C_6_–H_6_, G units (G)
H_2,6_	127.2/7.20	C_2,6_–H_2,6_ in H units (H)

**Table 6:** Structural characteristics of lignin by integration of ^13^C-^1^H correlation signals in the HSQC NMR spectrum of the ammonia-extractable lignin; lignin inter-unit linkages, percentage of γ-acylation, relative molar composition of the lignin aromatic units and S/G ratio.

Linkage relative abundance (% of total side chains involved)	Relative proportion (%)
*Lignin inter-unit linkages*	
β-Aryl-ether units (β-*O*-4′, A/A′)	75.6
Resinol substructures (β-β′, B)	12.2
Phenylcoumaran substructure (β-5′, C)	7.4
*p*-Hydroxycinnamyl alcohol end groups (F)	4.9
Percentage of γ-acetylation	0.8
*Lignin aromatic units*	
H (%)	1
S (%)	40
G (%)	59
S/G ratio	0.7

## References

[1] Mohammadi-Rovshandeh J., Sereshti H. (2005). The effect of extraction and prehydrolysis on the thermoplasticity and thermal stability of chemically modified rice straw. Iran. Polym. J.

[2] Ikeda T., Holtman K., Kadla J.F., Chang H.M., Jameel H. (2002). Studies on effect of ball milling on lignin structure using a modified DFRC method. J. Agric. Food Chem.

[3] Boerjan W., Ralph J., Baucher M. (2003). Lignin biosynthesis. Annu. Rev. Plant Biol.

[4] Rencoret J., Gutiérrez A., Nieto L., Jiménez-Barbero J., Faulds C.B., Kim H., Ralph J., Martínez Á.T., del Río J.C. (2011). Lignin composition and structure in young *versus* adult *Eucalyptus globulus* plants. Plant Physiol..

[5] Gellerstedt G., Henriksson G., Belgacem M.N., Gandini A. (2008). Lignins: Major sources, structure and properties. Monomers, Polymers and Composites from Renewable Resources.

[6] Ross K., Mazza G. (2010). Characteristics of lignin from flax shives as affected by extraction conditions. Int. J. Mol. Sci.

[7] Campbell M.M., Sederoff R.R. (1996). Variation in lignin content and composition. mechanisms of control and implications for the genetic improvement of plants. Plant Physiol.

[8] Vanholme R., Demedts B., Morreel K., Ralph J., Boerjan W. (2010). Lignin biosynthesis and structure. Plant Physiol.

[9] Sticklen M.B. (2008). Plant genetic engineering for biofuel production: towards affordable cellulosic ethanol. Nat. Rev. Genet.

[10] Weng J.K., Li X., Bonawitz N.D., Chapple C. (2008). Emerging strategies of lignin engineering and degradation for cellulosic biofuel production. Curr. Opin. Biotechnol.

[11] Mansfield S.D. (2009). Solutions for dissolution-engineering cell walls for deconstruction. Curr Opin. Biotechnol.

[12] Xu F., Sun R.C., Zhai M.Z., Sun J.X., Jiang J.X., Zhao G.J. (2008). Comparative study of three lignin fractions isolated from mild ball-milled *Tamarix austromogoliac* and *Caragana sepium*. J. Appl. Polym. Sci.

[13] Kondo R., Sako T., Limori T., Imamura H. (1990). Formation of glycosidic lignin-carbohydrate complex in the dehydrogenative polymerization of coniferyl alcohol. Mokuzai Gakkaishi.

[14] Guerra A., Filpponen I., Lucia L.A., Argyropoulos D.S. (2006). Comparative evaluation of three lignin isolation protocols for various wood species. J. Agric. Food Chem.

[15] Pye E.K., Kamm B., Gruber P.R., Kamm M. (2008). Industrial lignin production and applications. Biorefineries-Industrial Processes and Products.

[16] Kim H.S., Cho D.H., Won K., Kim Y.H. (2009). Inactivation of *Coprinus cinereus* peroxidase during the oxidation of various phenolic compounds originated from lignin. Enzyme Microb. Tech.

[17] Wang K., Xu F., Sun R.C. (2010). Molecular Characteristics of kraft-AQ pulping lignin fractionated by sequential organic solvent extraction. Int. J. Mol. Sci.

[18] Zhang A.P., Lu F.C., Liu C.F., Sun R.C. (2010). Isolation and characterization of lignins from *Eucalyptus tereticornis* (12ABL). J. Agric. Food Chem.

[19] Björkman A. (1957). Studies on finely divided wood. Part V. the effect of milling. Svensk Papperst.

[20] Bardet M., Robert D.R. (1985). On the reactions and degradation of the lignin during steam hydrolysis of aspen wood. Svensk Papperst.

[21] Ramos L.P. (2003). The chemistry involved in the steam treatment of lignocellulosic materials. Quim. Nova.

[22] Li J., Henriksson G., Gellerstedt G. (2007). Lignin depolymerization/repolymerization and its critical role for delignification of aspen wood by stream explosion. Bioresour. Technol.

[23] Sannigrahi P., Kim D.H., Jung S., Ragauskas A.J. (2011). Pseudo lignin and pretreatment chemistry. Energy Environ. Sci.

[24] Klinke H.B., Thomsen A.B., Ahring B.K. (2004). Inhibition of ethanol-producing yeast and bacteria by degradation products produced during pre-treatment of biomass. Appl. Microbiol. Biotechnol.

[25] Björkman A. (1954). Isolation of lignin from finely divided wood with neutral solvents. Nature.

[26] Xiao L.P., Shi Z.J., Xu F., Sun R.C. (2011). Characterization of MWLs from *Tamarix ramosissima* isolated before and after hydrothermal treatment by spectroscopical and wet chemical methods. Holzforschung.

[27] Zhang B., Huang H.J., Ramaswamy S. (2008). Reaction kinetics of the hydrothermal treatment of lignin. Appl. Biochem. Biotechnol.

[28] Wyman C.E., Dale B.E., Elander R.T., Holtzapple M., Ladisch M.R., Lee Y.Y. (2005). Coordinated development of leading biomass pretreatment technologies. Bioresour. Technol.

[29] Kim T.H., Kim J.S., Sunwoo C.S., Lee Y.Y. (2003). Pretreatment of corn stover by aqueous ammonia. Bioresour. Technol.

[30] Deng H., Lu J.J., Li G.X., Zhang G.L., Wang X.G. (2011). Adsorption of methylene blue on adsorbent materials produced from cotton stalk. Chem. Eng. J.

[31] Rencoret J., Marques G., Gutierrez A., Nieto L., Jimenez-Barbero J., Martinez A.T., del Rio J.C. (2009). Isolation and structural characterization of the milled-wood lignin from *Paulownia fortunei* wood. Ind. Crop. Prod.

[32] Fasching M., Schröder P., Wollboldt R.P., Weber H.K., Sixta H. (2008). A new and facile method for isolation of lignin from wood based on complete wood dissolution. Holzforschung.

[33] Scalbert A., Monties B. (1986). Comparison of wheat straw lignin preparations II, Straw lignin solubilisation in alkali. Holzforschung.

[34] Sun R.C., Lawther J.M., Banks W.B. (1996). Effects of extraction time and different alkalis on the composition of alkali soluble wheat straw lignins. J. Agric. Food Chem.

[35] Sun R.C., Sun X.F., Fowler P., Tomkinson J. (2002). Structural and physico-chemical characterization of lignins solubilized during alkaline peroxide treatment of barley straw. Eur. Polym. J.

[36] Faix O. (1991). Classification of lignins from different botanical origins by FT-IR spectroscopy. Holzforschung.

[37] Tai D.S., Terazawa M., Chen C.L., Chang H.M. (1990). Lignin biodegradation products from birch wood by phanerochaete chrysosporium. part I. fractionation of methanol-extractive and characterization of ether-insoluble low-molecular-weight fraction. Holzforschung.

[38] Li M.F., Fan Y.M., Sun R.C., Xu F. (2010). Characterization of extracted lignin of bamboo (*Neosinocalamus affinis*) pretreated with sodium hydroxide/urea solution at low temperature. Bioresources.

[39] Del R., Jose C., Rencoret J., Marques G., Li J.B., Gellerstedt G., Jiménez-Barbero J., Martínez A.T., Gutiérrez A. (2009). Structural characterization of the lignin from jute (*Corchorus capsularis*) fibers. J. Agric. Food Chem.

[40] Zhang X.M., Yuan T.Q., Peng F., Xu F., Sun R.C. (2010). Separation and structural characterization of lignin from hybrid poplar based on complete dissolution in DMSO/LiCl. Separ. Sci. Technol.

[41] Wen J.L., Sun Z.J., Sun Y.C., Sun S.N., Xu F., Sun R.C. (2010). Structural characterization of alkali-extractable lignin fractions from bamboo. J. Biobased Mater. Bioenergy.

[42] Lu H.F., Hu R.F., Ward A., Amidon T.E., Liang B., Liu S.J. (2012). Hot-water extraction and its effect on soda pulping of aspen woodchips. Biomass Bioenergy.

[43] Nguyen T.A.D., Kim K.R., Han S.J., Cho H.Y., Kim J.W., Park S.M., Park J.C., Sim S.J. (2010). Pretreatment of rice straw with ammonia and ionic liquid for lignocellulose conversion to fermentable sugars. Bioresour. Technol.

